# Escalating Antifungal Resistance Among *Candida* Species in Reproductive-Age Women in Vietnam: Implications for Women’s Health and Healthcare Systems

**DOI:** 10.3390/pathogens15060625

**Published:** 2026-06-11

**Authors:** Bac V. G. Nguyen, Tu Thien Nhat Nguyen, Bang Chau Ngoc Tu, Hung Van Cao, Bich Ngoc Thi Nguyen, Thanh Tri Vu, Gia-Phong Vu, Hoai Thu Le, Phuoc Vinh Nguyen

**Affiliations:** 1 School of Pharmacy, University of Medicine and Pharmacy at Ho Chi Minh City, Ho Chi Minh City 700000, Vietnam; nguyenvugiangbac@ump.edu.vn; 2 Faculty of Pharmacy, University of Health Sciences, Vietnam National University Ho Chi Minh City, Ho Chi Minh City 700000, Vietnam; nnttu@uhsvnu.edu.vn; 3 Faculty of Biology and Biotechnology, University of Sciences, Vietnam National University Ho Chi Minh City, Ho Chi Minh City 700000, Vietnam; tungocbangchau97@gmail.com; 4 Faculty of Medicine, University of Health Sciences, Vietnam National University Ho Chi Minh City, Ho Chi Minh City 700000, Vietnam; cvhung@uhsvnu.edu.vn; 5 Thu Duc General Hospital, Ho Chi Minh City 700000, Vietnam; dr.nobita1983@gmail.com (B.N.T.N.); drthanhtrinh2000@yahoo.com (T.T.V.); 6 Program in Comparative Biochemistry, University of California, Berkeley, CA 94720, USA; giaphongvu@berkeley.edu; 7 Center of Molecular Biomedicine, University of Medicine and Pharmacy at Ho Chi Minh City, Ho Chi Minh City 700000, Vietnam; lethuhoai@ump.edu.vn; 8 Research Center of Discovery and Development of Healthcare Products, Vietnam National University Ho Chi Minh City, Ho Chi Minh City 700000, Vietnam

**Keywords:** vulvovaginal candidiasis, *Candida albicans*, non-*albicans Candida*, pregnancy, antifungal resistance, Vietnam, PCR-RFLP

## Abstract

Vulvovaginal candidiasis (VVC) is a common fungal infection among reproductive-age women and is increasingly challenged by the emergence of non-*albicans Candida* species and reduced azole susceptibility. This prospective cross-sectional study investigated 235 symptomatic reproductive-age women attending two healthcare facilities in Ho Chi Minh City, Vietnam, to determine VVC prevalence, *Candida* species distribution, pregnancy-associated patterns, antifungal susceptibility, and diagnostic performance. Vaginal swabs were cultured on Sabouraud Dextrose Agar and CHROMagar™ Candida, while species identification was confirmed by PCR-RFLP targeting the ITS region. Susceptibility to fluconazole and clotrimazole was assessed using the disk diffusion method. *Candida* spp. was detected in 55.7% of participants. *C. albicans* accounted for 50.3% of isolates, whereas non-*albicans Candida* species represented 49.7%, indicating a substantial species shift. VVC was more frequent among pregnant women, particularly in the third trimester. Most *C. albicans*, *C. tropicalis*, and *C. parapsilosis* isolates remained susceptible to azoles; however, *C. glabrata* showed markedly reduced susceptibility to fluconazole and clotrimazole. CHROMagar™ Candida reliably identified *C. albicans* but misclassified several non-*albicans Candida* isolates compared with PCR-RFLP. These findings highlight the need for routine species-level diagnosis, antifungal susceptibility testing, and strengthened VVC surveillance in reproductive and antenatal healthcare settings in Vietnam.

## 1. Introduction

Vulvovaginal candidiasis (VVC) is among the most common fungal infections affecting women of reproductive age and remains a persistent clinical challenge in gynecological and reproductive healthcare. Globally, approximately 75% of women experience at least one episode of VVC during their lifetime, while 5–8% develop recurrent vulvovaginal candidiasis, a condition associated with repeated relapse, persistent symptoms, and increasing therapeutic difficulty [1,2]. Although VVC is often regarded as a non-life-threatening infection, its burden is clinically meaningful because recurrent or inadequately treated disease can lead to chronic vulvovaginal discomfort, impaired sexual and reproductive health, psychological distress, repeated healthcare visits, and reduced quality of life [3,4]. The clinical relevance of VVC is particularly important in low- and middle-income settings, where limited access to laboratory-based diagnosis and the frequent use of empirical therapy may delay appropriate treatment and contribute to persistent or recurrent infection.

The pathogenesis of VVC is driven by the transition of *Candida* species from commensal colonizers to opportunistic pathogens within the vaginal microenvironment. This transition is influenced by host, microbial, and environmental factors, including antibiotic exposure, immune dysregulation, hormonal changes, mucosal disruption, and alterations in the vaginal microbiota [5]. Pregnancy represents a particularly vulnerable physiological condition because elevated estrogen levels, increased vaginal glycogen deposition, changes in vaginal pH, and a shift toward Th2-dominant immunity create favorable conditions for fungal proliferation and persistence [6]. These pregnancy-associated changes may increase the risk of symptomatic VVC, recurrence, and treatment failure, especially during later gestational stages. International studies have reported VVC prevalence rates of up to 50% among pregnant women, with substantial geographical variation and higher rates frequently observed in tropical and subtropical regions [7]. In Vietnam, previous investigations reported VVC prevalence rates of 52.2% among women of reproductive age and 51.3% among symptomatic non-pregnant women, suggesting that VVC represents a substantial but still under-characterized reproductive health concern [8,9]. Similar high burdens have also been documented in pregnant women in other low-resource regions, including African settings, further supporting the need for locally adapted surveillance and management strategies [10]. More recently, regional evidence from Southeast Asia has also indicated that VVC remains an under-surveilled reproductive health problem with heterogeneous prevalence across countries, emphasizing the need for country-specific epidemiological and microbiological data [11].

A major emerging concern in VVC management is the changing etiological profile of infection. While *Candida albicans* has traditionally been the dominant causative species, increasing reports indicate a growing contribution of non-*albicans Candida* species, including *C. glabrata*, *C. krusei*, *C. tropicalis*, and *C. parapsilosis* [12]. This shift has important therapeutic implications because several non-*albicans Candida* species exhibit reduced susceptibility or intrinsic resistance to commonly used azole antifungals, especially fluconazole [13]. The clinical consequences are particularly serious in settings where empirical treatment remains common and species-level identification is not routinely performed. As a result, current treatment decisions may rely heavily on clinical symptoms and presumptive diagnosis, which may be inadequate in the context of increasing non-*albicans Candida* prevalence and azole resistance.

Accurate species identification is essential for effective VVC management because different *Candida* species vary substantially in pathogenicity, recurrence potential, biofilm-forming ability, and antifungal susceptibility. Conventional culture remains useful for fungal isolation, and Sabouraud Dextrose Agar continues to be widely used in clinical mycology laboratories; however, culture conditions can influence fungal recovery, growth characteristics, adhesion, and biofilm development, which may affect downstream interpretation [14]. CHROMagar™ Candida provides rapid presumptive identification based on colony morphology and color, but its discriminatory performance may be limited for several non-*albicans Candida* species. In contrast, molecular approaches such as PCR-RFLP targeting the internal transcribed spacer region offer improved species-level resolution and may be particularly useful in recurrent infections, pregnancy-associated VVC, and suspected antifungal resistance. These diagnostic improvements are increasingly important because resistance in *Candida* spp. is shaped by multiple mechanisms, including altered drug targets, efflux pump overexpression, stress-response adaptation, and biofilm-associated tolerance [13,15].

The therapeutic challenge is further intensified by the intrinsic and acquired resistance profiles of emerging non-*albicans Candida* species. Evidence from clinical populations has shown that *Candida* species differ markedly in antifungal susceptibility, reinforcing the need for local surveillance and species-guided treatment [16]. Azole resistance has been increasingly documented in *C. albicans* and emerging non-*albicans Candida* species, with mechanisms involving ERG11 alterations, upregulation of efflux transporters, and adaptive responses to antifungal exposure [17]. Biofilm formation on the vaginal mucosa may further reduce antifungal effectiveness and contribute to persistence or recurrence, particularly in mucosal infections where host–pathogen interactions are strongly influenced by local immune and hormonal factors [18]. Earlier species-identification studies also highlight that accurate differentiation of *Candida* isolates is clinically necessary because morphologically similar isolates may differ substantially in treatment response [19].

Despite the clinical importance of VVC, standardized diagnostic protocols, routine fungal screening in antenatal care, and antifungal resistance surveillance remain underdeveloped in Vietnam. This gap is concerning because delayed diagnosis, over-the-counter antifungal use, and empirical treatment may contribute to recurrence, mismanagement, and the selection of resistant strains. Therefore, updated local data are urgently needed to inform clinical decision-making, strengthen reproductive health programs, and guide antifungal stewardship. The present study aimed to determine the prevalence of VVC among symptomatic reproductive-age women in Vietnam, characterize the distribution of *Candida* species according to pregnancy status and gestational trimester, assess susceptibility to fluconazole and clotrimazole, and compare the diagnostic performance of CHROMagar™ Candida with PCR-RFLP. By integrating epidemiological, microbiological, diagnostic, and susceptibility data, this study provides updated evidence on the evolving burden of VVC in Vietnam and highlights the need for species-level diagnosis, routine susceptibility testing, and strengthened surveillance in reproductive and antenatal healthcare settings.

## 2. Materials and Methods

### 2.1. Sample Collection

This prospective cross-sectional study enrolled 235 women between 18 and 49 years old with complete data who visited the Thu Duc city Hospital, North of Ho Chi Minh City and Hung Phuc Clinics, Center of Ho Chi Minh City from October 2023 to January 2024.

The volunteers were interviewed one-on-one and those that gave consent to be part of the study were enrolled and who presented with symptoms of vaginal candidiasis, such as itching, burning, discharge and pain. The inclusion criteria included (1) having at least one of the above symptoms; (2) having no history of antifungal treatment in the past month.

After explaining the procedure and obtaining verbal consent, the clinician gently inserts a sterile speculum into the vagina without lubrication or with minimal water-based lubricant. Using a sterile cotton swab or wooden spatula, the clinician collects a sample of vaginal discharge from the posterior fornix or lateral vaginal walls. The specimen is then transferred onto a clean glass tube which contained 2 mL of 0.9% normal saline. Then, the clinician wrote the name and code for each sample, transferred them to our laboratory to begin the culture procedure.

Exclusion criteria included current menstruation, the use of systemic or topical antimicrobial or antifungal agents within the past month, or engagement in sexual intercourse, vaginal douching, and the use of vaginal hygiene products within 48 h prior to specimen collection. Additionally, individuals who were unable or unwilling to provide formal informed consent were excluded from the study.

### 2.2. Sample Screening

For primary screening, samples were cultured on Sabouraud Dextrose Agar (SDA) supplemented with 4% glucose and chloramphenicol. The high dextrose concentration in the medium promotes the growth of yeasts, particularly *Candida* species, while the addition of chloramphenicol inhibits bacterial contaminants without affecting fungal growth. This selective environment facilitates the isolation and proliferation of *Candida* spp. by suppressing competing bacterial growth. Specimens were directly inoculated onto the surface of the SDA plates using sterile cotton swabs and incubated at 37 °C for 48 to 72 h. *Candida* colonies typically appeared as white or cream-colored patches, allowing for preliminary identification and assessment of fungal presence.

### 2.3. Identification of Candida Species by CHROMagar Candida

From the initial growth on Sabouraud Dextrose Agar (SDA), one to three colonies were aseptically collected using a sterile inoculation loop and streaked onto CHROMagar™ Candida plates (Himedia, Maharashtra, India). The inoculated plates were then incubated at 37 °C for 48 to 72 h. Following incubation, colony morphology and color were observed and compared with the manufacturer’s reference guide to presumptively identify the *Candida* species.

### 2.4. Molecular Identification of Candida Species

A modified DNA extraction method adapted from Wahyuningsih et al. was employed to isolate genomic DNA from *Candida* colonies. Initially, a loopful of yeast cells was collected from the surface of a Sabouraud Dextrose Agar (SDA) plate and suspended in 100 μL of sterile deionized water. Subsequently, 455 μL of a lysis buffer composed of 240 mM sodium hydroxide (NaOH) 2.7 mM ethylenediaminetetraacetic acid (EDTA), and 74% ethanol was added to the suspension. The mixture was incubated at 80 °C for 10 min to promote effective cell lysis, followed by centrifugation at 13,200 rpm for 10 min. After discarding the supernatant, the resulting pellet was resuspended in 100 μL of extraction buffer consisting of 50 mM Tris-HCl (pH 8.0), 0.1 mM EDTA, 1% Triton X-100 (Merck, Rahway, NJ, USA), and 0.5% Tween 20. The suspension was mixed thoroughly to ensure uniform dispersion and centrifuged at 12,000 rpm for 15 min. The aqueous phase, which contained the extracted DNA, was carefully transferred to a new sterile microcentrifuge tube.

Quantification of DNA was performed using a UV–Visible spectrophotometer (GeneQuant 1300, Biochrom, Holliston, MA, USA), and each sample was subsequently diluted to a working concentration of 200 ng/μL for molecular analyses.

Amplification of the internal transcribed spacer (ITS) region was carried out using the primer pair ITS1 (5′-TTC GTA GGT GAA CCT GCG G-3′) and ITS4 (5′-TCC TCC GCT TAT TGA TAT GC-3′), which target conserved fungal sequences. The PCR reaction mixture (25 μL total volume) included 17.3 μL of NF-Q1 solution, 2.5 μL of iTag buffer, 1 mM dNTPs, 250 μM of each primer, 100 ng of genomic DNA, and 1.0 U of Taq DNA polymerase. Thermal cycling was performed using a SimpliAmp Thermal Cycler (Thermo Fisher Scientific, Waltham, MA, USA), programmed for 30 cycles with an annealing temperature of 53 °C, an extension time of 40 s.

Post-amplification, the PCR products were subjected to digestion with the restriction enzyme *Hpa*II (0.15 U/μL) at 37 °C for 15 min to generate species-specific restriction fragment profiles. The digested products were resolved via electrophoresis on a 10% polyacrylamide gel using a 100 bp Opti-DNA ladder (Applied Biological Materials Inc., Richmond, BC, Canada) as a molecular size marker. Visualization was performed using a UV transilluminator (TFX-20 LC, Vilber Lourmat, France). Isolates were identified by comparing the observed banding patterns with known ITS fragment sizes and digestion profiles characteristic of different *Candida* species.

### 2.5. Determine the Susceptibility of Isolated Candida Species

To evaluate the antifungal susceptibility of the isolated *Candida* species to fluconazole and clotrimazole, the disk diffusion method was performed in accordance with the Clinical and Laboratory Standards Institute CLSI M44 guideline for antifungal disk diffusion susceptibility testing of yeasts [20]. Briefly, standardized fungal suspensions were prepared from fresh *Candida* colonies and adjusted to the appropriate turbidity. The suspensions were then uniformly inoculated onto Mueller–Hinton agar supplemented with glucose and methylene blue. Antifungal disks containing fluconazole and clotrimazole were placed on the inoculated agar surface, and the plates were incubated at 35–37 °C for 24–48 h. After incubation, the inhibition zone diameters around each disk were measured in millimeters and interpreted according to CLSI M44 criteria, where applicable, to classify isolates as susceptible, intermediate/susceptible-dose dependent, or resistant. This standardized procedure enabled reproducible assessment of antifungal susceptibility profiles among the clinical *Candida* isolates.

### 2.6. Statistical Analysis

Data were summarized using descriptive statistics. The distribution of *Candida* species and antifungal susceptibility profiles was presented as frequencies and percentages. Because the study primarily aimed to describe the number and distribution of clinical *Candida* isolates.

### 2.7. Ethical Aspects

All sampling procedures constructed in the current study were reviewed and accepted by the ethics committee of the University of Health Sciences (authorized by the Ministry of Health of Vietnam, IRB-VN01.017) under decision No (05/KHSK-HDDD/GCN).

## 3. Results

### 3.1. Prevalence and Clinical Characteristics of Vaginitis Among Reproductive-Age Women

Analysis revealed that women in their second trimester accounted for a significant proportion (44.6%) of reproductive-age women presenting with symptoms of vaginitis (Table 1). Overall, the prevalence of vaginitis was higher among pregnant women (59.5%) compared to non-pregnant women of reproductive age. The most frequently reported symptom was profuse vaginal discharge, observed in 100% of symptomatic cases, followed by the presence of thick, white clumps adhering to the vaginal walls without odor (92.7%). Due to the non-severe and non-specific nature of these symptoms, many women overlook it or resort to self-medication with over-the-counter antifungal agents rather than seeking medical care.

**Figure 1:**
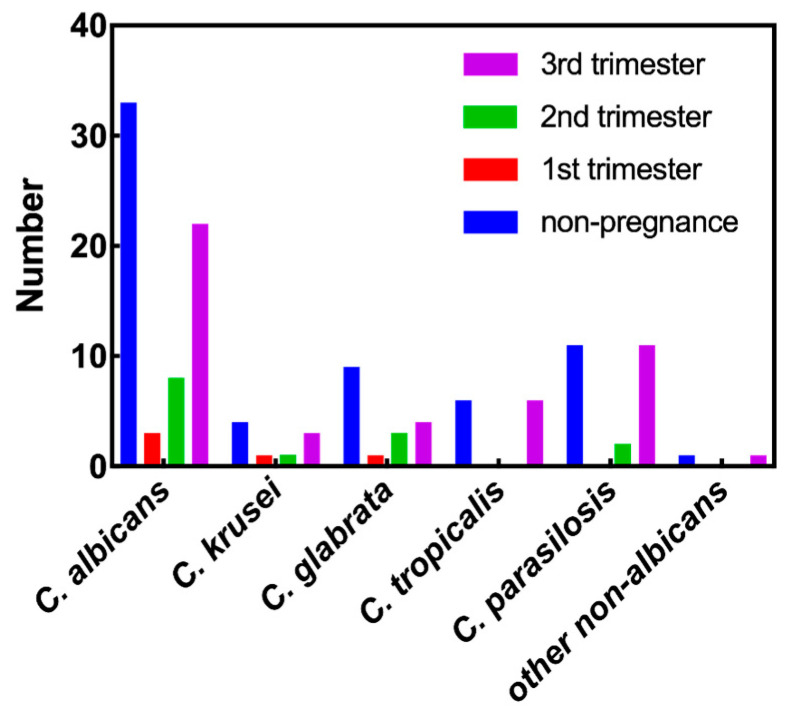
Prevalence of Candida species in Vietnamese reproductive women.

The prevalence of *Candida* spp. in this study was 55.74%, notably higher than the 35.3% reported by Le Hoai Chuong in 2013 [8] and slightly elevated compared to the 46.8% reported by Do Ngoc Anh in 2020 [9]. These findings may reflect a growing burden of vulvovaginal candidiasis among women in Vietnam, especially in pregnancy, underscoring the need for routine screening and targeted public health interventions (Figure 1).

Analysis of the *Candida* species distribution in women of reproductive age, stratified by pregnancy status and trimester, reveals a striking trend: a pronounced surge in VVC among women in their third trimester. While *Candida* infections were found in both non-pregnant and pregnant women, a detailed breakdown shows that the third trimester accounts for the highest burden of infection, comprising 59.6% of all cases among pregnant women.

This rise is not trivial—it reflects an alarming shift in susceptibility as pregnancy advances. During the first trimester, infections were relatively rare, representing only 5 isolates (10.6% of total cases in pregnancy), and increased modestly in the second trimester with 14 isolates (29.8%). However, by the third trimester, the number of infections surged to 28 cases. The distribution of *Candida* isolates across pregnancy trimesters showed a progressive increase, with the highest number of isolates observed in the third trimester. However, this finding should be interpreted as a descriptive observation rather than evidence of a statistically confirmed increasing trend, as the study was not specifically powered for trimester-stratified comparisons.

Among the *Candida* species identified, *C. albicans* remains the most prevalent, with 22 infections reported in the third trimester alone. This indicates that even though *C. albicans* is typically part of the normal vaginal flora, the changes in the host environment during late pregnancy may trigger its transformation from a benign colonizer to a pathogenic organism.

The situation is compounded by the emergence of NAC such as *C. glabrata*, *C. krusei*, and *C. parapsilosis*, which collectively contributed to a substantial number of cases in the third trimester. These species are not only harder to identify using conventional culture-based methods but also often exhibit reduced susceptibility or resistance to common azole antifungals like fluconazole and clotrimazole, posing significant challenges for empirical treatment. Of particular concern is *C. glabrata*, which was detected in 3 women in their third trimester and is well-documented to possess intrinsic resistance mechanisms, including reduced ergosterol affinity and efflux pump overexpression.

### 3.2. Comparison of Primary and Alternative Methods for Candida spp. Identification

Sabouraud Dextrose Agar (SDA) supplemented with chloramphenicol is widely recognized as the gold standard for the primary isolation of *Candida* species from clinical specimens due to its ability to selectively support fungal growth while suppressing bacterial contaminants. SDA provides a nutrient-rich environment that facilitates the recovery of viable fungal colonies, making it a reliable medium for initial screening and culture-based diagnostics [14].

**Figure 2:**
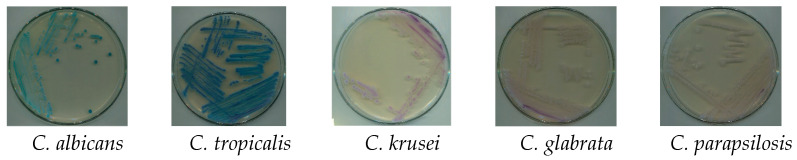
Morphological aspects of isolated fungal strains on CHROMagarTM.

However, in recent years, alternative or complementary approaches such as CHROMagar™ Candida and molecular methods like PCR-RFLP have been increasingly employed in clinical mycology. CHROMagar Candida, a chromogenic medium, not only supports fungal growth but also enables presumptive differentiation of *Candida* species based on colony color and morphology (Figure 2). This visual distinction facilitates early species-level identification directly from the culture plate, thereby streamlining the diagnostic workflow. Similarly, PCR-RFLP targeting the internal transcribed spacer (ITS) region of fungal rDNA offers high sensitivity and specificity in species determination, particularly in cases involving mixed infections or morphologically ambiguous isolates (Table 2).

Given their capabilities, both CHROMagar Candida and PCR-RFLP can serve as effective standalone tools for direct identification of *Candida* spp., potentially obviating the need for an initial culture on SDA. While SDA remains a reliable medium for fungal isolation, the combination or substitution with rapid identification methods such as CHROMagar or PCR-RFLP can enhance diagnostic efficiency, particularly in high-throughput or resource-constrained settings. Therefore, laboratories may consider implementing CHROMagar Candida and/or PCR-RFLP directly in diagnostic protocols for *Candida* identification, without the prerequisite of prior culture on SDA, provided that appropriate specimen handling and quality control measures are in place.

Biochemical identification of *Candida* species using CHROMagar™ Candida remains highly accurate for *Candida albicans*, with a concordance rate of up to 99.99% when compared with molecular identification methods. However, the reliability of CHROMagar decreases substantially when applied to non-*albicans Candida* (NAC) species. In contrast, PCR-RFLP targeting the internal transcribed spacer (ITS) region—amplified using ITS1 and ITS4 primers followed by digestion with species-specific restriction enzymes—has demonstrated high accuracy and reproducibility, making it a robust method for species-level identification in clinical microbiology laboratories.

In this study, notable discrepancies were observed between biochemical and molecular identification for NAC species. Especially, discordance rates were 18.75% for *C. tropicalis*, 30% for *C. glabrata*, 67.8% for *C. krusei*, 100% for *C. parapsilosis*, and 86.67% for other uncommon *Candida* species. These findings indicate that CHROMagar Candida, while useful for presumptive identification, may lead to species misclassification among NAC isolates, potentially impacting treatment decisions due to differing antifungal susceptibility profiles.

Therefore, while CHROMagar remains a practical and cost-effective screening tool, confirmatory testing using PCR-RFLP is recommended for accurate identification, especially in settings where NAC species are frequently encountered or when precise speciation is critical for guiding antifungal therapy.

### 3.3. Drug Susceptibility of Isolated Candida Species to Fluconazole and Clotrimazole

As showed in Table 3, both fluconazole and clotrimazole demonstrated relevant antifungal activity against the majority of *Candida* species tested, particularly against *C. albicans* (90.9% and 86.36%, respectively). In addition, a high susceptibility to fluconazole and clotrimazole was observed in *C. parapsilosis* (87.5% and 95.83%) and *C. tropicalis* (83.33% and 91.67%, respectively). For *C. krusei*, the susceptibility was lightly decreased, with 77.8% to fluconazole and 66.67% to clotrimazole. Interestingly, other less common non-*albicans Candida* species showed complete susceptibility (100%) to both antifungal agents.

In contrast, *C. glabrata* exhibited markedly reduced susceptibility to both drugs, with only 29.4% of isolates responding to fluconazole and 12.5% to clotrimazole. These findings are consistent with previous reports indicating that *C. glabrata* possesses intrinsic or acquired resistance mechanisms to azole antifungals, posing significant clinical challenges in treatment.

Taken together, the results highlight the continued efficacy of fluconazole and clotrimazole for most *Candida* species, while emphasizing the need for species-specific identification and antifungal susceptibility testing, especially in cases involving *C. glabrata*, to ensure appropriate therapeutic management.

### 3.4. Drug Susceptibility of Candida Species Across Pregnancy Stages

**Figure 3:**
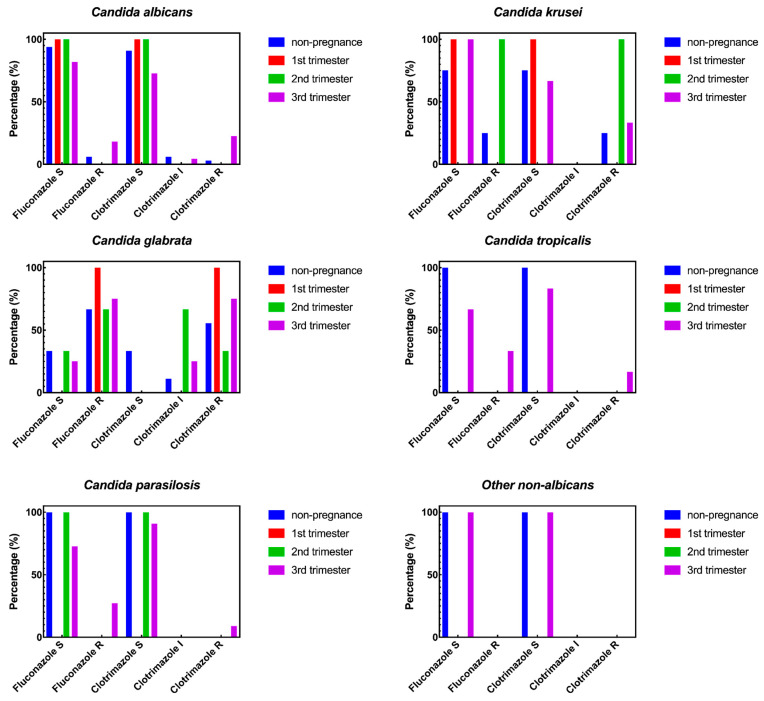
Drug susceptibility of *Candida* species across pregnancy stages in Vietnamese women.

The antifungal susceptibility results reveal a concerning trend of increased resistance to both fluconazole and clotrimazole among *Candida* species, particularly in the later stages of pregnancy (Figure 3). In the non-pregnant population, susceptibility to fluconazole was relatively high across most species, with *C. albicans*, *C. tropicalis*, and *C. parapsilosis* showing 93.9%, 100%, and 100% susceptibility, respectively. Clotrimazole also demonstrated strong efficacy in this group, with *C. albicans* at 90.9%, *C. tropicalis* and *C. parapsilosis* both maintaining full sensitivity.

However, this pattern shifts notably as pregnancy progresses. In the third trimester—a physiologically vulnerable period characterized by heightened hormonal, immunological, and mucosal changes—multiple *Candida* species show a clear decrease in antifungal susceptibility. For *C. albicans*, susceptibility to fluconazole dropped to 81.8% and clotrimazole to 72.7%, accompanied by an increase in resistance (fluconazole: 18.2%; clotrimazole: 22.7%). These changes are particularly concerning given that *C. albicans* remains the dominant pathogen responsible for vulvovaginal candidiasis.

The situation is more alarming with *C. glabrata*, which displayed consistently low susceptibility across all trimesters. In the third trimester, only 25% of isolates were sensitive to fluconazole, while 75% were resistant. Clotrimazole performed slightly better, but resistance remained significant. This highlights the intrinsic or rapidly acquired resistance of *C. glabrata*, making it a species of special concern, especially in late pregnancy when treatment options become more limited due to potential drug-related fetal risks.

Similarly, *C. krusei* presented mixed responses. While 100% of isolates were susceptible to fluconazole in the first trimester, susceptibility declined to 66.7% in the third trimester. Clotrimazole also showed reduced efficacy, with one-third of isolates being resistant by the third trimester. These fluctuations suggest that pregnancy may not only influence fungal colonization but also impact the resistance profiles of these pathogens, possibly through altered host-microbe dynamics or repeated self-medication with over-the-counter antifungals, which can drive resistance.

*C. tropicalis* and *C. parapsilosis*, while initially highly susceptible, also exhibited waning drug sensitivity in the third trimester. For *C. parapsilosis*, fluconazole susceptibility dropped from 100% in non-pregnant women to 72.7% in the third trimester, and clotrimazole susceptibility declined to 90.9%. Although still relatively effective, these figures reflect a tangible loss in drug potency and demand closer monitoring in late pregnancy.

Although reduced susceptibility to fluconazole and clotrimazole was observed among several third-trimester isolates, particularly *C. glabrata*, these findings should be interpreted cautiously because of the limited number of isolates within each species and pregnancy subgroup. Therefore, the observed patterns should be considered preliminary and hypothesis-generating rather than conclusive evidence of trimester-associated antifungal resistance. Larger multicenter studies using MIC-based susceptibility methods are needed to validate these observations.

## 4. Discussion

### 4.1. Epidemiological Importance and Contextual Trends

The detection of *Candida* spp. in 55.7% of symptomatic Vietnamese women underscores a significant public health concern, especially when placed against previous Vietnamese reports such as 52.2% by Do Thi Thuy Dung et al. [8] and 51.3% by Do Ngoc Anh et al. [9]. This notable increase suggests a potential upward trend in *Candida* prevalence over the past decade. Environmental factors such as high humidity, increasing antibiotic and antifungal self-medication, and a lack of public health awareness may be driving this trend. Additionally, the findings highlight an under-recognized burden of vaginal fungal infections in low-to-middle-income Southeast Asian countries, where routine gynecological screening is often absent.

The current data are consistent with global estimates ranging from 30 to 60%, with tropical and subtropical climates consistently associated with higher prevalence [7]. The present study not only strengthens the epidemiological profile of VVC in Vietnam but also provides a foundational baseline for future longitudinal studies and surveillance programs targeting women’s health.

### 4.2. Species Distribution and a Shift from C. albicans into NAC Species

While *C. albicans* continues to be the most frequently isolated species (50.3%), our study identified a growing trend toward NAC species including *C. glabrata*, *C. parapsilosis*, *C. tropicalis*, and *C. krusei*, accounting for nearly 50% of isolates. This shift is not isolated to Vietnam—it has been documented globally in settings such as the U.S., China, and South America [11,15].

The increased prevalence of NAC species is particularly concerning due to their reduced sensitivity to azole antifungals and unique pathogenic mechanisms. For example, *C. glabrata* and *C. krusei* exhibit intrinsic or acquired resistance to fluconazole, often necessitating alternative regimens such as echinocandins or boric acid suppositories [16,17]. Additionally, NAC infections are more likely to be asymptomatic or present with mild symptoms, increasing the risk of misdiagnosis and delayed treatment.

This shift in the etiological profile has critical clinical implications: empirical treatment protocols based solely on *C. albicans* susceptibility profiles may no longer be effective in up to half of VVC cases. A renewed emphasis on species-level identification, either through advanced culture methods or molecular diagnostics, is warranted to guide accurate treatment.

### 4.3. Impact of Pregnancy on Drug Susceptibility

One of the most significant contributions of this study is the documentation of *trimester-specific* susceptibility to VVC, with markedly higher rates in the third trimester. This observation is supported by immunophysiological changes during pregnancy that predispose women to *Candida* colonization. Rising estrogen levels stimulate glycogen accumulation in vaginal epithelial cells, creating an ideal substrate for fungal growth, while the Th2 immune shift weakens antifungal host defenses [4,18].

Clinically, VVC during late pregnancy is not benign. It has been linked to adverse obstetric outcomes including preterm labor, low birth weight, and neonatal candidiasis through vertical transmission [3,10]. These risks justify the implementation of routine screening for VVC during antenatal care visits, particularly in the second and third trimesters—a practice currently lacking in most Vietnamese maternity protocols.

Although pregnancy-related hormonal and immunological changes may increase susceptibility to *Candida* colonization and symptomatic VVC, the present study did not directly assess the mechanistic relationship between hormonal status and antifungal resistance. Therefore, the observed increase in resistance among third-trimester isolates should be interpreted cautiously. This pattern may reflect pregnancy-associated host factors, prior antifungal exposure, species composition, or other unmeasured variables. Further studies incorporating hormonal profiling, detailed treatment history, and molecular resistance analysis are needed to clarify these associations.

Similarly, self-medication with over-the-counter antifungal agents may contribute to antifungal selection pressure at the population level; however, this study did not systematically evaluate self-medication behavior beyond excluding participants with recent antifungal treatment. Thus, self-medication should be considered a possible explanatory factor rather than a confirmed determinant of resistance in our cohort. Future behavioral and pharmacoepidemiological studies are warranted to better assess the relationship between antifungal use patterns and resistance development in VVC.

### 4.4. Drug Susceptibility Patterns and Resistance Mechanisms

Our antifungal susceptibility testing confirmed that while *C. albicans*, *C. parapsilosis*, and *C. tropicalis* remain largely sensitive to fluconazole and clotrimazole, *C. glabrata* exhibited alarming resistance—only 25% of isolates were susceptible. These findings are in line with global resistance patterns and underline the need for continuous resistance monitoring [1,11].

Mechanistically, *C. glabrata* and *C. krusei* possess multiple resistance strategies, including overexpression of efflux pumps (CDR1, CDR2, MDR1), ERG11 gene mutations, and adaptive biofilm formation [12]. These mechanisms complicate treatment, especially in resource-limited settings where echinocandins are unavailable or unaffordable.

Furthermore, increased antifungal resistance in third-trimester isolates of even *C. albicans* may point toward hormonal modulation or biofilm enhancement in late pregnancy. These observations warrant further investigation through transcriptomic and in vitro biofilm assays [19].

Although the disk diffusion method is simple, inexpensive, and suitable for routine screening, it has important limitations compared with MIC-based methods such as broth microdilution or gradient diffusion assays. Disk diffusion only provides categorical susceptibility results based on inhibition zone diameters and does not generate exact MIC values. As a result, it may be less sensitive in detecting subtle reductions in susceptibility, dose-dependent susceptibility, or emerging low-level resistance. In addition, the results may be influenced by technical variables, including inoculum density, agar thickness, disk potency, incubation conditions, and zone-reading interpretation. Therefore, the susceptibility results in this study should be interpreted as screening data. Future studies should incorporate MIC-based methods to provide more quantitative and clinically informative antifungal susceptibility profiles, particularly for NAC species with known azole resistance potential.

### 4.5. Diagnostic Limitations and Recommendations

Despite the widespread use of CHROMagar™ Candida for rapid presumptive identification, this medium misidentified several NAC species in our study, with particularly poor specificity for *C. parapsilosis* and *C. glabrata*. This misidentification may lead to inappropriate antifungal choice and prolonged infection.

Conversely, PCR-RFLP targeting the ITS region offered robust species-level discrimination, even among closely related NAC strains. This technique should be considered for diagnostic upgrades in tertiary hospitals and antenatal clinics managing recurrent or resistant VVC cases. However, its implementation in primary care remains limited by cost and infrastructure.

Although this study demonstrated the diagnostic advantages of CHROMagar™ Candida and PCR-RFLP compared with SDA culture for *Candida* species identification, the direct application of these methods to clinical specimens without prior culture was not evaluated. Future studies should investigate direct-from-sample molecular identification workflows to further reduce diagnostic turnaround time and improve clinical applicability.

### 4.6. Public Health and Policy Implications

The findings of this study carry considerable public health relevance. First, the data call for enhanced VVC surveillance, particularly in high-risk groups such as pregnant women. Second, there is an urgent need to regulate over-the-counter azole antifungal distribution to reduce self-medication and resistance emergence. Public health campaigns focusing on early symptom recognition, the importance of gynecological check-ups, and adherence to treatment guidelines are critical [11].

At the policy level, integrating fungal screening into national antenatal protocols—especially during the second and third trimesters—could reduce maternal-fetal transmission and improve perinatal outcomes. Additionally, subsidizing antifungal diagnostics and alternative treatments (e.g., boric acid, nystatin) would provide broader access to effective therapy in rural and underserved populations.

### 4.7. Contributions and Future Directions

This is the first comprehensive Vietnamese study to (i) map *Candida* species distribution stratified by pregnancy trimester, (ii) assess antifungal resistance trends, and (iii) evaluate diagnostic performance of both phenotypic and molecular techniques. Our findings offer a critical framework for developing updated VVC management guidelines in Southeast Asia.

Future studies should aim to include larger multi-center data, longitudinal monitoring of resistance patterns, and whole-genome sequencing of resistant strains. Integrating microbiological data with clinical outcomes will further refine treatment pathways and improve women’s health across the region.

## 5. Conclusions

This study highlights a high prevalence of *Candida* infections among women of reproductive age presenting with symptoms of vulvovaginal candidiasis (VVC) in Ho Chi Minh City, Vietnam. *Candida albicans* remained the dominant species; however, non-*albicans Candida* species—known for their variable antifungal susceptibility—were frequently isolated, indicating a potential shift in species distribution that requires clinical attention. The observed species-specific differences in antifungal susceptibility, particularly the reduced sensitivity of *C. glabrata* to both fluconazole and clotrimazole, underscore the need for accurate identification and tailored antifungal therapy.

The comparison with previous national and international studies confirms both geographical variation and an increasing trend in non-*albicans Candida* prevalence, which may be associated with host factors, over-the-counter antifungal use, and suboptimal antimicrobial stewardship. These findings emphasize the importance of continuous epidemiological surveillance, incorporation of molecular diagnostic methods for species identification, and regular antifungal susceptibility testing to inform effective treatment guidelines and reduce the emergence of resistance.

In conclusion, addressing the clinical and microbiological dynamics of VVC in Vietnam requires a multifaceted approach, combining laboratory vigilance with public health interventions, patient education, and evidence-based prescribing practices to ensure optimal management and prevent future therapeutic failure.

## Figures and Tables

**Table 1:** Symptoms of Candidiasis amongst reproductive women according to birthage.

Women in Birthage	The Vaginal Area Is Red, Itchy and Painful	The White Blood Clumps into Pieces, Forming Thick Patches that Stick to the Walls of the Underworld, Without Odor	A Lot of Vaginal Discharge	Frequent Urination, Difficulty Urinating
0	3	93	95	2
0–3	7	19	24	5
3–6	9	98	105	7
>6	2	8	14	4
n%	8.9%	92.7%	100%	7.7%

**Table 2:** Prevalence of *Candida* species identified using morphological and molecular methods.

*Candida* spp.	Chromagar *Candida*	PCR-RFLP
*Candida albicans*	65	66
*Candida krusei*	28	9
*Candida glabrata*	7	17
*Candida tropicalis*	16	12
*Candida parapsilosis*	0	24
Different *non-albicans*	15	2

**Table 3:** Drug susceptibility of isolated *Candida* species to fluconazole and clotrimazole.

*Candida* spp.	Fluconazole			Clotrimazole		
	S	R	I	S	R	I
*Candida albicans*	60	6		57	6	3
*Candida krusei*	7	2		6	3	
*Candida glabrata*	5	12		3	10	4
*Candida tropicalis*	10	2		11	1	
*Candida parapsilosis*	21	3		23	1	
Different *Non-Candida albicans*	2			2		

## Data Availability

All data generated or analyzed during this study are included in this article. Further enquiries can be directed to the corresponding author.

## Abbreviations

The following abbreviations are used in this manuscript:

VVC	Vulvovaginal candidiasis
ITS	Internal transcribed spacer
SDA	Sabouraud Dextrose Agar
NAC	Non-albicans Candida

## References

[1] Denning D.W., Kneale M., Sobel J.D., Rautemaa-Richardson R. (2018). Global burden of recurrent vulvovaginal candidiasis: A systematic review. Lancet Infect. Dis..

[2] Sobel J.D. (2007). Vulvovaginal candidosis. Lancet.

[3] Dong Z., Fan C., Hou W., Rui C., Wang X., Fan Y., Zhao L., Wang Q., Wang Z., Zeng X. (2021). Vaginal Exposure to Candida albicans During Early Gestation Results in Adverse Pregnancy Outcomes via Inhibiting Placental Development. Front. Microbiol..

[4] Juliana N.C.A., Suiters M.J.M., Al-Nasiry S., Morré S.A., Peters R.P.H., Ambrosino E. (2020). The Association Between Vaginal Microbiota Dysbiosis, Bacterial Vaginosis, and Aerobic Vaginitis, and Adverse Pregnancy Outcomes of Women Living in Sub-Saharan Africa: A Systematic Review. Front. Public Health.

[5] Achkar J.M., Fries B.C. (2010). Candida infections of the genitourinary tract. Clin. Microbiol. Rev..

[6] Fidel P.L. (2002). Immunity to Candida. Oral Dis..

[7] Bitew A., Abebaw Y. (2018). Vulvovaginal candidiasis: Species distribution of Candida and their antifungal susceptibility pattern. BMC Womens Health.

[8] Dung D.T.T., Anh D.N., Hoa H.T., Yen N.T.H. (2022). Determining the Composition of Fungi in Female Patients with Genital Inflammation to Be Examined and Treated at Military HospitaL 103. J. Nurs. Sci..

[9] Anh D.N., Hung D.N., Tien T.V., Dinh V.N., Son V.T., Luong N.V., Van N.T., Quynh N.T.N., Van Tuan N., Tuan L.Q. (2021). Prevalence, species distribution and antifungal susceptibility of Candida albicans causing vaginal discharge among symptomatic non-pregnant women of reproductive age at a tertiary care hospital, Vietnam. BMC Infect. Dis..

[10] Osman Mohamed A., Suliman Mohamed M., Hussain Mallhi T., Abdelrahman Hussain M., Ali Jalloh M., Ali Omar K., Omar Alhaj M., Makki Mohamed Ali A.A. (2022). Prevalence of vulvovaginal candidiasis among pregnant women in Africa: A systematic review and meta-analysis. J. Infect. Dev. Ctries..

[11] Falcon R.M.G., Alcazar R.M.U., Guda J.V., Tantengco O.A.G. (2024). A systematic review and meta-analysis on the prevalence of vulvovaginal candidiasis in Southeast Asian countries. SciEnggJ.

[12] Pappas P.G., Kauffman C.A., Andes D.R., Clancy C.J., Marr K.A., Ostrosky-Zeichner L., Reboli A.C., Schuster M.G., Vazquez J.A., Walsh T.J. (2016). Clinical Practice Guideline for the Management of Candidiasis: 2016 Update by the Infectious Diseases Society of America. Clin. Infect. Dis..

[13] Cowen L.E., Sanglard D., Howard S.J., Rogers P.D., Perlin D.S. (2014). Mechanisms of Antifungal Drug Resistance. Cold Spring Harb. Perspect. Med..

[14] Weerasekera M.M., Wijesinghe G.K., Jayarathna T.A., Gunasekara C.P., Fernando N., Kottegoda N., Samaranayake L.P. (2016). Culture media profoundly affect *Candida albicans* and *Candida tropicalis* growth, adhesion and biofilm development. Mem. Inst. Oswaldo Cruz.

[15] Perlin D.S. (2015). Echinocandin resistance in *Candida*. Clin. Infect. Dis..

[16] Maheronnaghsh M., Fatahinia M., Dehghan P., Teimoori A. (2020). Identification of *Candida* species and antifungal susceptibility in cancer patients with oral lesions in Ahvaz, Southern West of Iran. Adv. Biomed. Res..

[17] Whaley S.G., Berkow E.L., Rybak J.M., Nishimoto A.T., Barker K.S., Rogers P.D. (2017). Azole antifungal resistance in *Candida albicans* and emerging non-*albicans Candida* species. Front. Microbiol..

[18] Harriott M.M., Lilly E.A., Rodriguez T.E., Fidel P.L., Noverr M.C. (2010). *Candida albicans* forms biofilms on the vaginal mucosa. Microbiology.

[19] Baradkar V.P., Kumar S. (2009). Species identification of *Candida* isolates obtained from oral lesions of HIV infected patients. Indian J. Dermatol..

[20] (2018). Method for Antifungal Disk Diffusion Susceptibility Testing of Yeasts, 3rd ed.

